# The Risk of COVID-19 Infection in Prisons and Prevention Strategies: A Systematic Review and a New Strategic Protocol of Prevention

**DOI:** 10.3390/healthcare10020270

**Published:** 2022-01-29

**Authors:** Massimiliano Esposito, Monica Salerno, Nunzio Di Nunno, Federica Ministeri, Aldo Liberto, Francesco Sessa

**Affiliations:** 1 Department of Medical, Surgical and Advanced Technologies “G.F. Ingrassia”, University of Catania, 95121 Catania, Italy; massimiliano.esposito91@gmail.com (M.E.); monica.salerno@unict.it (M.S.); federicaministeri@gmail.com (F.M.); aldoliberto@gmail.com (A.L.); 2 Department of History, Society and Studies on Humanity, University of Salento, 73100 Lecce, Italy; nunzio.dinunno@unisalento.it; 3 Department of Clinical and Experimental Medicine, University of Foggia, 71122 Foggia, Italy

**Keywords:** prison, COVID-19, prevention, risk of infection, management strategies

## Abstract

Health risks within prisons are well known and have worsened with the 2019 coronavirus pandemic (COVID-19), becoming a public health emergency. To date, there are more than 10 million inmates in the world; in most cases, conditions are bad and health care is scarce. A SARS-CoV-2 outbreak inside a prison is extremely rapid. The aim of this systematic review was to analyze all possible prevention techniques to reduce the risk of COVID-19 related infection within prisons. A systematic review of the literature was performed according to the PRISMA guidelines. Scopus, Web of Science, PubMed, and Google Scholar were used as search engines from 1 January 2020 to 1 November 2021 to evaluate the prevention of COVID-19 in prisoners. A total of 1757 articles were collected. Of them, 486 duplicates were removed. A total of 1250 articles did not meet the inclusion criteria. In conclusion, 21 articles were included in the present systematic review. From this analysis, it emerged that the most common COVID-19 prevention methods were the screening of the entire population (prisoners and workers) inside the prison through swab analysis and the reduction in overcrowding in prisons. Few studies concerned the prevention of COVID-19 infection through vaccination and the implementation of quarantine. To our knowledge, this is the first systematic review that evaluates the prevention of COVID-19 within jails and the real effectiveness of all possible methods used and published in the literature. Finally, a very useful strategic protocol is provided to reduce the incidence of infection and to control and manage COVID-19 in prisons.

## 1. Introduction

Health risks inside prisons, particularly in overcrowded and under-resourced ones, are well known. However, with the severe acute respiratory syndrome coronavirus 2 (SARS-CoV-2) pandemic in 2019 (COVID-19), they became a public health emergency [1]. To date, in fact, there are more than 10 million prisoners around the world; in most cases, the conditions are bad and health care is poor [2]. The spacing in the cells is difficult to manage. Failure to control infection in prisons would also pose a risk to the general population. Furthermore, the increase in restrictions inside prisons could lead to a worsening of prisoners’ human rights [3]. In the pandemic period, the risk of infection within prisons has been strictly related to different factors such as overcrowding, level of education, and medical and environmental conditions [4]. It is wrong to think that, since the detainees are already in isolation from the rest of the world, the hazard of COVID-19 infection is lower [5]. In fact, the general population believes that because prisons are a closed environment and prisoners do not leave jail, inmates are less exposed in prison. However, the influx of people from outside is constant inside a prison, for example, the staff (guards, cleaners, etc.), lawyers, and family members. Thus, once a single prisoner is infected, in no time an outbreak could develop. SARS-CoV-2 infection can develop rapidly within prisons, increasing its incidence, morbidity, and mortality [6]. The World Health Organization (WHO) has published guidelines on the prevention of infections within prisons, supporting the importance of the use of personal protective equipment (PPE), social distancing, and prisoners’ mental health [7]. The release of prisoners is a highly debated topic: on the one hand, it could reduce the overcrowding of prisons, and on the other hand, it would put prisoners on probation who may not have a home to live in. This could lead to an increase in crime. For this reason, some countries have tried to implement hygiene rules inside prisons [8]. Another countermeasure used inside prisons is the use of throat swabs and serological tests for the detection of COVID-19 as a screening method, not only for all prisoners but also for those who work in the prison (policemen, doctors, nurses, administrators, etc.) [9,10,11]. Finally, vaccination could also be a useful tool to prevent the spread of COVID-19 infection in prisons. In fact, to date, vaccination is the most effective means of prevention and treatment against COVID-19, reducing incidence, morbidity, hospitalization, and mortality [12,13,14,15,16]. However, studies on the effectiveness of the vaccination campaign in prisons are few and hopefully will be implemented in the future. A recent survey stated that 80% of inmates are in favor of a vaccination campaign inside the jails. Even despite these preventive and restrictive measures, the infection rate for SARS-CoV-2 is still very high, with serious repercussions on the mental health of prisoners [17]. In fact, the present systematic review collects all the prevention strategies within prisons and aims to create a single strategy capable of effectively limiting COVID-19 in prisons.

The aim of this systematic review was to analyze all possible prevention techniques to reduce the risk of COVID-19 related infection within prisons. As far as we are aware, this is the first systematic review that evaluates the prevention of COVID-19 within jails and the real effectiveness of all possible methods used and published in the literature. A further aim of the present review is to suggest a very useful strategic protocol to reduce the incidence of infection and to prevent COVID-19 infection in prisons. This protocol could be used by Prison Directors as a helpful tool to refer to, not only to reduce SARS-CoV-2 infection, but also for all other infections that are difficult to manage in prisons (tuberculosis, HCV, HIV, etc.).

## 2. Materials and Methods

A systematic review of the literature was performed according to the PRISMA guidelines [18].

Scopus, Web of Science, PubMed, and Google Scholar were used as search engines from 1 January 2020 to 1 November 2021 to evaluate the prevention of COVID-19 in prisoners. The following keywords were used: (prison OR jail) AND (COVID OR sars); (prison OR jail) AND (risk of infection OR hazard of infection) AND (COVID OR sars); (prison OR jail) AND (risk of infection OR hazard of infection).

### 2.1. Inclusion and Exclusion Criteria

The exclusion criteria were: (1) wrong publication type (articles not relevant to the study), (2) review, (3) letters or editorials, (4) articles not in English, (5) meta-analysis. The inclusion criteria were: (1) original article, (2) survey, (3) articles regarding the risk of COVID-19 infection, (4) articles in English.

### 2.2. Quality Assessment and Data Extraction

M.E. and F.S. analyzed all the articles, evaluating the entire text. In cases of discrepancy of opinions between inclusion or exclusion of articles, they were submitted to M.S.

### 2.3. Characteristics of Eligible Studies

**Figure 1:**
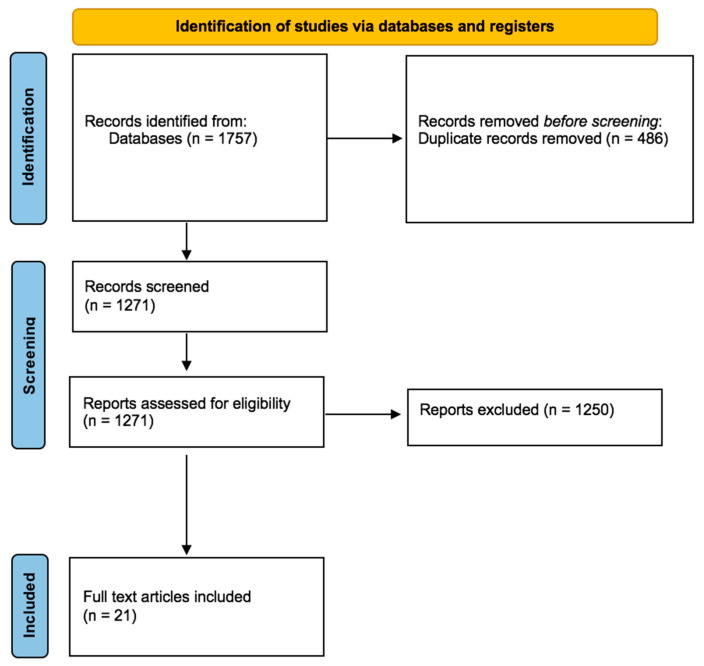
Flow diagram illustrating included and excluded studies in this systematic review.

A total of 1757 articles were collected. Of these, 486 duplicates were removed. A total of 1250 articles did not meet the inclusion criteria. Therefore, 21 articles were included in the present systematic review (Figure 1) [19,20,21,22,23,24,25,26,27,28,29,30,31,32,33,34,35,36,37,38,39].

## 3. Results

Of the 21 studies included, most were observational cohort studies (40%) and ecological studies (35%), and the rest were retrospective cohort and cross-sectional studies. In most cases (60%), the studies were conducted in prisons in the United States of America (USA), with the rest in Central South America (Brazil, Guatemala, Mexico) and in Europe, such as United Kingdom (UK), Italy, and Ireland. The most common COVID-19 prevention methods were the screening of the entire population (prisoners and workers) inside the prison through swab analysis and the reduction of overcrowding in prisons. In particular, the overcrowding of prisons was guaranteed through the regulated release of prisoners with minor offenses, the reduction of visits to inmates, the reduction of prison transfers, and the decrease in the number of people inside the cell. Few studies concerned the prevention of COVID-19 infection through vaccination and the implementation of quarantine. A study conducted in Italy used both the serological test for the detection of IgM and IgG of COVID-19 and the analysis of throat swabs [23].

Regulated release worked best in overcrowded prisons to reduce the risk of infection. Reinhart, E. et al. [35] claimed that controlled decarceration reduced the infection rate by eight times in overcrowded prisons, while the use of masks reduced the infection rate by 2.5%, while the visit ban reduced the infection rate by 1.2%. Brinkley-Rubinstein, L. et al. [30] concluded that inter-prison transfers reduced the risk of COVID-19 infection. Furthermore, the incidence of COVID-19 was lower in prisons where there was an adequate number of prisoners and in those in which detainees were housed in single-cell units [34].

Clarke, M. et al. [26] used the contact tracing team (CTT). Specifically, in cases of a suspected COVID-19 prisoner, the CTT was notified and analyzed all the contacts of this suspected case. All of these prisoners were subjected to both swabs and subsequently quarantine, regardless of the outcome of the swab. If one prisoner had a positive result, the cycle was repeated. If the prisoner was negative, the quarantine continued but the cycle of contact tracing was interrupted.

Obviously, all hygiene measures and the use of PPE were also essential for reducing the hazard of COVID-19 infection.

Finally, all of the included studies stated that the risk of COVID-19 infection in prisons was higher than in the general population, thus, prevention measures were needed to reduce the risk of transmission. In fact, Marquez, N.M. et al. [31] calculated a mortality in 2020 that was 42% higher than that of 2019, of which 80.4% was due to COVID-19. Jiménez, M.C. et al. [25] affirmed that the COVID-19 rate among incarcerated individuals was nearly three times that of the general population of Massachusetts and five times the rate in the United States. Only one study showed the effects of vaccination within prisons. Brinkley-Rubinstein et al. [32] stated that there were few studies evaluating COVID-19 and vaccination in prisons. Specifically, 2380 residents inside the prison (prisoners and staff) who had received at least one dose of vaccine were analyzed. Only 27 had tested positive for SARS-CoV-2, testifying to the vaccine’s effectiveness even in the prison population. The authors concluded that the vaccine was an extremely effective tool for the prevention of COVID-19 in prisons. Table 1 summarizes the evidence from this systematic review.

## 4. Discussion

The prison population is an extremely vulnerable population [40]. There are 2.3 million people in prisons and juvenile facilities in the USA. It is estimated that in the USA the incarceration rate is equal to 698 prisoners per 100,000 inhabitants (24% of all prisoners worldwide), and the outbreak of COVID-19 made inmates particularly vulnerable [41,42,43]. In Brazil, deaths in jails accounted for 17.5% of all deaths, with a peak in Rio de Janeiro, where it was 28.5%. With the related COVID-19 pandemic, this risk has increased even more, and the Brazilian government has suspended prisoner transfers and prison visits; however, this has not slowed the incidence of the virus [44].

The COVID-19 entry routes into prisons may be different. At the beginning of the pandemic a COVID-19 entry route into prisons came from the external environment. Visitors (e.g., family members) can be a vehicle for transmitting the infection, and visits were prohibited in many countries [45,46,47]. Even the staff (e.g., cleaners, policemen, etc.), for the same reason, can be a vector of infection. In fact, initially, the incidence of COVID-19 is higher among prison staff than among prisoners. However, subsequently, the situation reversed and outbreaks among prisoners were almost unmanageable, resulting in the need for infection prevention measures [48]. In fact, the SARS-CoV-2 epidemic inside a prison is extremely fast; it was estimated that after only 30 days from the start of the pandemic, 7.8% of staff and 5.6% of prisoners were already positive for throat swab for SARS-CoV-2 [49,50]. Shen et al. [51] claims that a positive COVID-19 subject within a cell has a 60% chance of infecting the other resident of the cell. Obviously, if one inmate lives in a dormitory, the other inmate can be infected at the same time, especially considering the poor ventilation inside prisons.

An observational study evaluated the outcome of COVID-19 disease in prisoners compared to the general population. This study showed that inmates were more frequently symptomatic (fever, tachypnea, hypoxemia) and were more commonly admitted to the intensive care unit (ICU) and intubated. Finally, in-hospital mortality was more frequent in prisoners than in the general population [52]. Saloner et al. [53] calculated that the incidence of COVID-19 inside prisons was 5.5 times higher; in some facilities, prisoners had been swab screened for SARS-CoV-2 with a prevalence of 65% of positives for the total number of prisoners. The correlated COVID-19 death rate in prisons was 39 deaths per 100,000 prisoners, while in the general population it was 29 deaths per 100,000 people.

These results are consistent with those of the present systematic review. In fact, Blair, A. [19] tested some Canadian prisoners through swabs by PCR analysis for SARS-CoV-2 and found 29% positives compared with 6% of the general population (non-prisoners); mortality was 0.6%. Marquez et al. [31], on the other hand, through a retrospective cohort study using data reported by the Florida Department of Corrections, stated that, comparing mortality in prisons from 2015 to 2019 with that of 2020, mortality in 2020 was 42% higher, with 80.4% deaths related to COVID-19.

Social distancing is often recommended, but it is almost always difficult to maintain inside prisons. In the absence of recommendations from state governments, many prisons acted independently and were unable to prevent infection, developing outbreaks. According to some authors [54], action should be taken on three levels: macro level by raising the awareness of state governments; medium level through unique protection policies in cities; and at the micro level through the single detention center. The recommendations provided by the Centers for Disease Control and Prevention (CDC) [55] include the administration of mass tests through PCR obtained by means of swabs inside prisons, regardless of the presence of symptoms. The asymptomatic or pre-symptomatic subjects make up 40–45% of the totality of COVID-19 positive prisoners. The same recommendations also include the administration of SARS-CoV-2 PCR-search swabs to staff members at regular intervals, regardless of the presence of symptoms. According to Quan et al. [56], however, releasing ex-inmates can amplify the transmission of SARS-CoV-2 when they pass to the general society. Therefore, viral screening through the use of oral-pharyngeal swabs can detect infected subjects who are in an asymptomatic or pre-symptomatic phase at the time of release. This could be an effective strategy to reduce the contagion. According to an article published in the American Journal of Public Health, the prevention policy within the prison should include several measures such as: early decarceration for minor offenses, improving the ventilation of common spaces and cells, using PPE appropriately, limiting transfers of prisoners to different structures, encouraging sanitation in prisons by improving health care, and providing mental health support [57,58]. However, the early release of prisoners in a pandemic state could cause difficulties for ex-prisoners in their rehabilitation within society. This could lead to a relapse in their delinquency or, for the weaker, frail, and elderly inmates, a sort of abandonment to society [59].

In China and in South Korea, on the other hand, various preventive measures have been adopted to deal with epidemic outbreaks inside prisons. First, they have funding for the purchase of adequate PPE. Second, they saw that many outbreaks originated from the staff and spread among the prisoners, so they set up shift work for the staff. Third, they created several areas for confirmed cases and suspected COVID-19 cases [60,61]. In Australia, on the other hand, the government adopted several bans against the development of epidemic outbreaks: suspension of visits between prisoners; decrease in transfers between different prisons; temperature control for staff at the entrance; introduction of quarantine periods for new prisoners; and creation of isolation hubs for positive prisons [62,63]. Prisons in the Philippines and Pakistan have a serious problem of prison overcrowding, and it is estimated that only 25% of inmates use face masks as PPE, so the Philippine government arranged to use CTTs as a model to prevent outbreaks within prisons [64,65]. In Italy, the Ministry of Justice and Health has implemented the practices of isolating prisoners and prisons from the outside world, and the creation of a sort of CTT for the identification of suspected or confirmed COVID-19 cases. However, this measure dramatically reduced prisoners’ human rights and only slightly slowed the progression of COVID-19 within prisons [66,67]. Italy also adopted other types of maneuvers to prevent COVID-19 infection within prisons, such as the possibility of home detention in cases of prisoners with less than 18 months of sentence to serve [68,69,70]. However, the release of prisoners is a highly debated topic: on the one hand, it could reduce overcrowding in prisons, but on the other hand, it would put prisoners on probation who may not have a home to live in. This could lead to an increase in crime. For this reason, some countries have tried to implement hygiene rules inside prisons [71]. In UK prisons, it has been estimated that the isolation of new inmates for 10–14 days systematically reduces the likelihood of new infections within the prison [72]. In Turkey, on the other hand, the prevention strategy is essentially based on the disinfection and decontamination of the prison environment; the suspension of all collective activities; the suspension of education, work, and training activities for prisoners; increase in PPE; 14-day quarantine for new inmates; and isolation of all suspected cases [73]. The Brazilian National Penitentiary also used the same measures, implementing PPE, freeing the elderly with serious chronic disease, reducing the number of inmates inside the cells, and suspending visits inside the prison [74]. Obviously, the situation in the poorest countries is much more serious; in fact, the prisons are almost all overcrowded, PPE is in short supply, and the risk of continuing epidemic outbreaks is extremely high. COVID-19 in these countries’ prisons is virtually uncontrollable [75]. According to Brelje et al. [76], to tackle COVID-19 inside prisons, it is necessary to increase the means of screening within these structures. Symptom screening alone could cause a subtle spread of the infection, so it must be supplemented with laboratory techniques (serum and swabs). In addition, fair distribution of all PPE for residents (staff and inmates) should be encouraged. Finally, ensure access to mental health care and support for all prisoners. A case report, on the other hand, reiterates the importance of health care during the COVID-19 pandemic; clinicians should ensure the health of all patients, especially if in prison, as they live in solitude. Specifically, a 73-year-old man with heart failure, chronic kidney failure, and diabetes mellitus was admitted to a hospital near the prison due to COVID-19 related pneumonia. Throughout the hospitalization, the prisoner was intubated and remained chained to the bed; after a few days he died in complete solitude, chained, losing his human rights [77].

The findings of this systematic review are consistent with other studies. Most of the included studies, in fact, concerned the screening of internal residents (prisoners and staff) of prisons through throat swabs. This method not only allowed the detection of symptomatic positives, but also asymptomatic and pre-symptomatic subjects, blocking potential epidemic outbreaks [19,22,37,38]. Pagano et al. [23] used both PCR swab analysis and serological testing, which were equally effective. Controlled decarceration was a very useful element in reducing the incidence of COVID-19 in prisons. Reinhart et al. [35] stated that controlled decarceration reduced the infection rate by eight times in overcrowded prisons. The use of masks reduced the infection rate by 2.5%, while the visit ban reduced the infection rate by 1.2%. Another effective measure was the CTT [26]: specifically, in cases of a suspected COVID-19 prisoner, the CTT was notified and analyzed all the contacts of this suspected case. All of these prisoners were subjected to both swabs and, subsequently, quarantine, regardless of the outcome of the swab. If a subject was positive, the cycle was repeated. If a subject was negative, quarantine continued but the cycle of contact tracing was interrupted. All studies also analyzed the risk of repercussions of these measures on the mental health of prisoners, confirming a strong concern for this aspect.

To date, there are few studies that analyze the effects of the vaccination campaign inside prisons. Prisons should not be seen in isolation from the global community, but as an integral part. Since the efficacy of the COVID-19 vaccine has been established, and since inmates are a fragile population with high mortality, vaccination should be a priority in prisons. Vaccination, in fact, represents an effective weapon to reduce epidemic outbreaks, morbidity, and related COVID-19 mortality of inmates inside prisons [78,79].

In fact, in the present study, only one study showed the effects of vaccination within prisons. Brinkley-Rubinstein et al. [32] stated that there were few studies evaluating COVID-19 in prisons and vaccination. Specifically, 2380 residents inside the prison (prisoners and staff) who had received at least one dose of the vaccine were analyzed. Only 27 tested positive for SARS-CoV-2, testifying the vaccine’s effectiveness even in the prison population. The authors concluded that the vaccine was an extremely effective tool for the prevention of COVID-19 in prisons.

**Figure 2:**
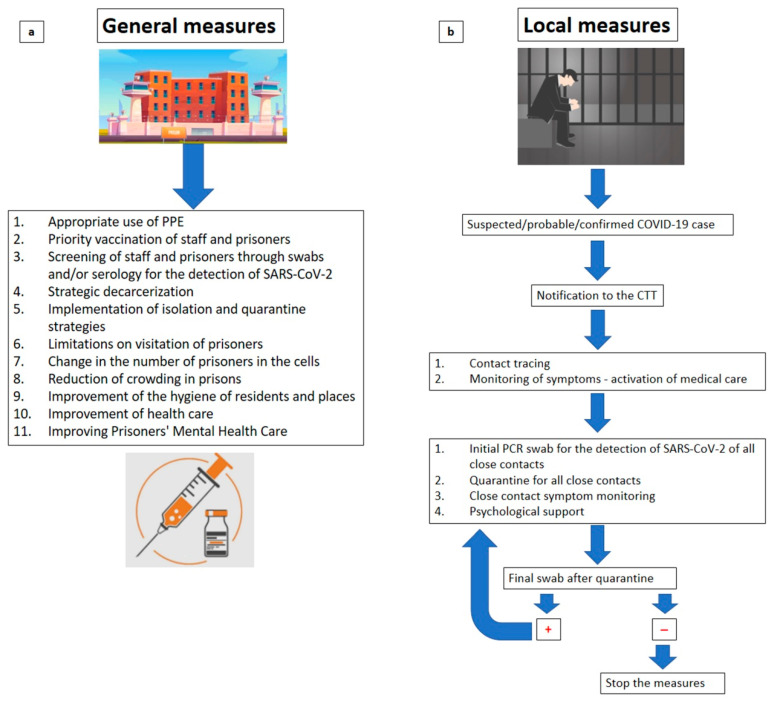
COVID-19 prevention measures in prisons in general (**a**) and within the single prison (**b**).

This study is, to date, the first systematic review that highlights the main aspects of COVID-19 in prisons, showing the incidence, morbidity, and mortality within these structures and providing the most important prevention techniques to reduce the risk of infection in prisons. Through this review, a preventive strategy for the risk of contagion is provided through the application of general measures to be implemented in prisons (improvement of environmental hygiene, appropriate use of PPE, release for minor crimes, mass screening, vaccination), and local measures in cases of a positive COVID-19 subject occurring within the prison (Figure 2a,b) [72,73,74,77].

Due to the fragility of people in prison and the precarious clinical condition of many prisoners, COVID-19 vaccination should be a priority in this population [17,80]. However, the vaccination campaign in prisons is still slow and incomplete, and should be encouraged by governments [81,82,83,84,85,86,87,88,89]. COVID-19 vaccination certification, also called “COVID-19 passport” or “Green Pass” (GP), exists in many countries [86,89]. Practically, the use of this certification is used indoors, for traveling, for gyms, and for entering shops—anywhere people gather. The purpose of the GP is to allow access to these places only to those who possess it, in order to limit the spread of COVID-19, an objective that today is still an effective weapon in the battle against SARS-CoV-2 [87].

However, this systematic review shows that the use of the GP in prisons is highly debated and not unique, and visitors to prisons (family members of inmates, lawyers) are not required to show COVID-19 vaccination certification in all countries. Initially, the WHO established that it was necessary to perform a check of the conditions for all visitors inside prisons [7]. In Italy, the government established that all visitors must not show a GP at the entrance to the prison [90]. In New Zealand, on the other hand, from 9 December 2021, all prison visitors aged 12 and over must be fully vaccinated and show their GP upon arrival [91]. In South Australia, the same provision was established in October 2021 [92]. In Ireland, visitors are not required to exhibit the GP, however, the government itself says this decision is under review [93]. In the USA, all visitors must complete a vaccination certification form stating that they are fully vaccinated, or submit a negative COVID-19 test result from an approved test performed within three days before entry [94].

**Figure 3:**
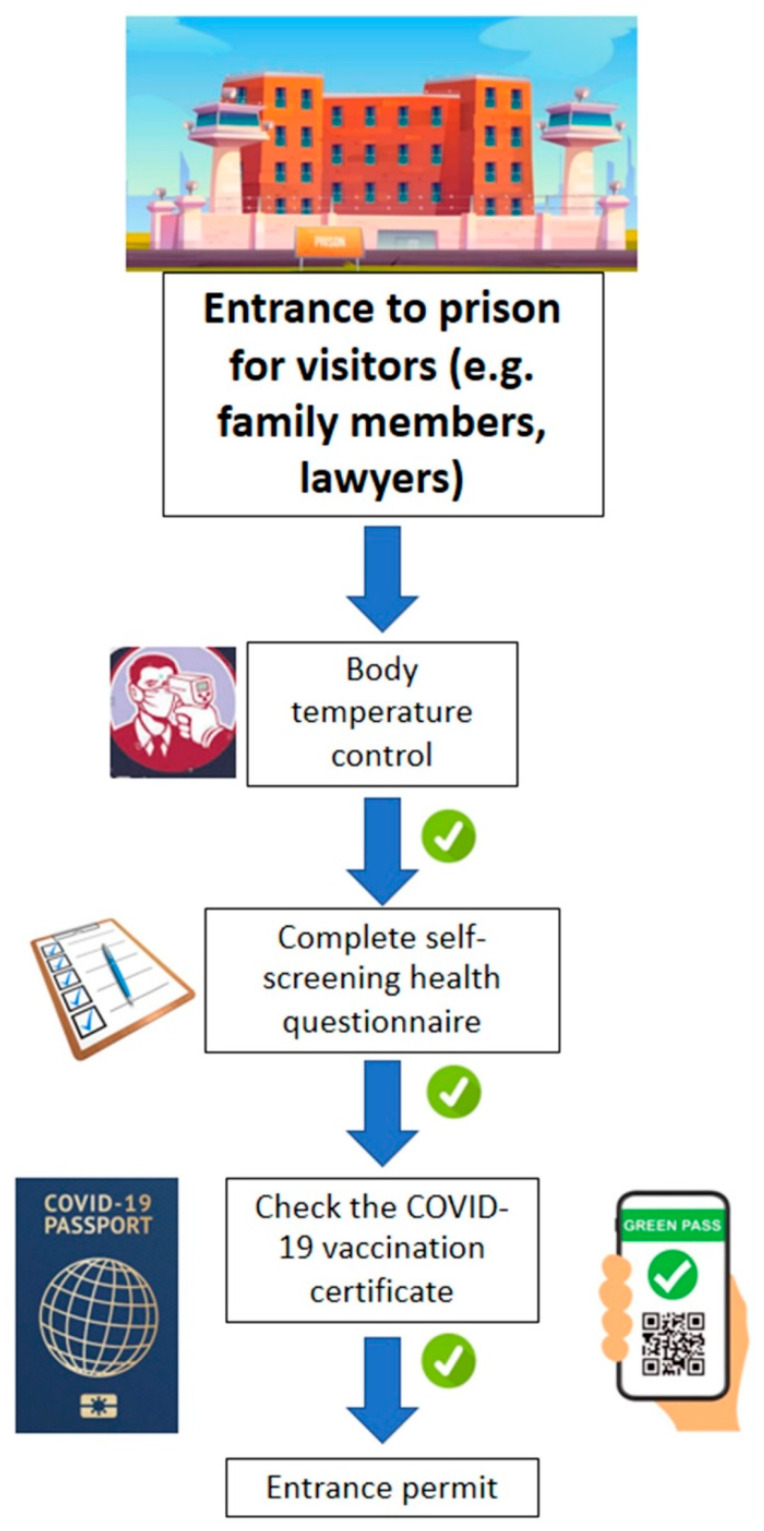
Prevention measures for COVID-19 for visitor in prisons.

In any case, the crucial role of the GP in the prevention of COVID-19 infection and the promotion of the vaccination campaign is undoubted. However, in the present study, it is shown that not all countries have adopted this measure in prisons, and there is much debate on the subject. This systematic review proposes a new model that is useful to further reduce the risk of COVID-19 outbreaks within prisons, which is based on reducing the risk of transmission of SARS-CoV-2 from outside by visitors. This review involves checking body temperature at the entrance to the prison, filling out a health questionnaire, and checking the GP (Figure 3).

## 5. Conclusions

The prisoner population is a highly vulnerable population, suffering from high rates of mental disorders, substance abuse disorders, poor medical conditions, old age, poorly hygienic dormitories, overcrowded facilities, high rate of infectious diseases, morbidity, and mortality [95,96]. Initially, the policy adopted to reduce the transmission of COVID-19 was to quarantine new inmates and the replenishment of PPE. However, these measures have often contributed to worsening the mental health of prisoners, thus, measures that ensure the psychological and physical well-being of this category are needed [97,98,99,100]. In fact, it must always be borne in mind that too strict regulations can cause damage to the mental health of prisoners; therefore, when implementing anti-COVID-19 measures, it is necessary to keep this aspect in mind [101,102]. Prisons should evaluate and create innovative strategies to promote prisoners’ mental health, including the establishment of periodic psychological therapies. Prisoners and all health care workers should work closely together to ensure health and infection prevention within these penitentiary facilities and provide adequate follow-up plans and periodic telemedicine appointments [103]. As noted by this systematic review, new studies about the risk of COVID-19 infection in prisons through the Green Pass Policy (GPP) should be encouraged. In fact, by comparing these results with those in which the GPP is not applied, it is possible to verify whether the GPP is effective in reducing the risk of COVID-19 infection in penitentiaries.

### Proposed Strategy

As clarified in Figure 2, “general measures” and “local measures” need to be implemented in a prison. General measures are those that should be implemented within a prison, such as the appropriate use of a filtering face piece 2 (FFP2s), an effective vaccination campaign, population screening by swabs and serology for SARS-CoV-2, implementation of safety measures, isolation and quarantine, reduction in the number of prisoners in cells, and improvement of the health and hygiene of all prisoners. By “local measures”, we mean those to be implemented in cases of prisoner positivity. In cases of suspected positivity, it is necessary to notify the CTT, then carry out a contact tracing operation and monitoring of symptoms. It is necessary to implement quarantine and a molecular swab for SARS-CoV-2 of all close contacts, together with psychological support. The cycle must be repeated if the result is still positive in the molecular control buffer.

Figure 3, on the other hand, clarifies the measures to be implemented for all people who access prisons. It is necessary to perform the following: a body temperature check, a questionnaire on health status, and a check of the COVID-19 vaccination certificate.

## Figures and Tables

**Table 1:** Summary of the details of the systematic review.

Reference	Study Design	Location of the Prison	Prevention Methods	Results	Conclusion
Blair et al. [19]	Observational cohort study	Canada	Swab analyses	29% of the prisoners tested were positive for swab by polymerase chain reaction (PCR) analysis for SARS-CoV-2, compared with 6% of the general population (non-prisoners). Mortality was 0.6%	Prison settings were very vulnerable to COVID-19 infection; therefore, swabs should be carried out not only for symptomatic patients but for all at-risk or suspected positive prisoners
Pitts et al. [20]	Cross-sectional study	Honduras, El Salvador, and Guatemala	In the prisons of Honduras, nearly 2000 prisoners were released in the early stages of the infection. In the prisons of El Salvador and Guatemala, they implemented the restrictive measures inside the prison	Reduction in virus transmission compared to prisons in neighboring countries	Prisoners were at greater risk of developing the virus than the general population; however, preventive measures and the release of prisoners with lesser sentences were useful measures to reduce the transmission of COVID-19
Marquez et al. [31]	Retrospective cohort study	Florida	Strategic decarceration for prisoners and priority vaccination for all prisoners	An analysis of mortality in prisons was performed, using data reported by the Florida Department of Corrections, comparing mortality from 2015 to 2019 with that of 2020. Mortality in 2020 was 42% higher with 80.4% deaths related to COVID-19	A significant increase in all-cause mortality was initially observed within Florida prisons during the period of the COVID-19 pandemic, leading to a decrease in life expectancy of more than four years
Parsons et al. [33]	Observational cohort study	California	Quarantine protocols	The risk of infection represented by the transfer of prisoners between the different prisons was examined. It was estimated that quarantine and isolation systems more effectively controlled the risk of COVID-19 infection within prisons than vaccination and decarceration	Quarantine and isolation measures were the most effective preventive measures to reduce the risk of COVID-19 infection
Leibowitz et al. [34]	Ecological study	Massachusetts	Reduction of crowding in prisons	The incidence of COVID-19 was lower in prisons where there were an adequate number of prisoners and in those in which detainees were housed in single-cell units	COVID-19 mortality and incidence increased in proportion to the overcrowding of prisons. Rescheduling of prisoner numbers reduced the risk of infection
Reinhart et al. [35]	Observational cohort study	51% of all the prisons in the USA	Limitations on visitation rights for prisoners, use of masks, controlled release for prisoners with minor offenses	Controlled decarceration reduced the infection rate by 8 times in overcrowded prisons. The use of masks reduced the infection rate by 2.5%, while the visit ban reduced the infection rate by 1.2%	To prevent the risk of COVID-19 contagion in prisons, controlled decarceration for minor crimes was a useful method to reduce infection, especially for overcrowded jails. However, wearing masks and banning visits decreased the risk of infection, too
Zeveleva et al. [36]	Ecological study	47 European countries	Strategic decarceration for prisoners, limitations on visitation rights for prisoners	All European states had implemented visit bans. Only 16 countries opted for the early release of prisoners with minor offenses in their prisons. Compared to the visit ban, the early release required more time and rules to be implemented. Early release was very useful in overcrowded prisons	The risk of COVID-19 infection in prisons is very high, the prohibition of visiting prisoners and/or their early release helped in decreasing the risk of contagion inside the prisons of 47 European states
Toblin et al. [37]	Ecological study	Federal Bureau of Prisons	Swab analyses	Mortality rates were higher in prisons where molecular PCR tests were not performed for the diagnosis of COVID-19 infection and reached up to 3%	Strategies of serial swab testing for COVID-19 within prisons decreases the risk of infection
Lemasters et al. [38]	Observational cohort study	Illinois, Maryland, Minnesota, New Mexico, and Virginia	Swab analyses	The more the prisoners were subjected to PCR swab analysis for COVID-19 the more the positive rate increased, reaching a rate of 42% in some prisons (such as Louisiana)	Prison had a higher prevalence of COVID-19 positives than the general population with increased risk of infection. However, using serial swabs for inmates could reduce the risk of SARS-CoV-2 infection
Chin et al. [39]	Ecological study	California	Change in the number of prisoners in individual jails	Overcrowded prisons had a higher positive rate, especially those who lived inside the dorms	By decreasing the number of people inside individual jails, the risk of COVID-19 infection decreased
Chan et al. [21]	Retrospective cohort study	New York city	Swab analyses	Of 978 prisoners tested for COVID-19, 568 were positive on swab analysis. Of the 568, 58 inmates were asymptomatic. Older age and diabetes mellitus increased the likelihood of hospitalization	The use of COVID-19 swab screening campaigns should be used to reduce the infection rate within prisons
Gouvea-Reis et al. [22]	Ecological study	Brazil	Swab analyses and reduction of crowding in prisons	Increase in COVID-19 contagion in overcrowded prisons	Social distancing was difficult to implement inside prisons, especially if they were overcrowded. The implementation of screening strategies in prisons was an effective method of preventing the risk of contagion
Pagano et al. [23]	Observational cohort study	Salerno (Italy)	Serum and swab analyses	COVID-19 serum screening was performed on all inmates. In doubtful results, a throat swab was performed in the shortest possible time. Out of 485 tests, 0.61% were positive	The application of mass screening for COVID-19 inside prisons (serological and swab in doubtful cases) reduced the risk of contagion
Wilburn et al. [24]	Retrospective cohort study	UK	Swab analyses	Of 1156 prisoners, 58 showed COVID-19 symptoms; of these, 62.1% tested positive for swab. The remainder was subjected to screening swabs for 5 consecutive days, always reporting negative results	The throat swab screening campaign was a very useful preventive tool
Jiménez et al. [25]	Ecological study	Massachusetts	Swab analyses	14,987 people were incarcerated in Massachusetts facilities. 664 were the COVID-19 swab positive prisoners. The COVID-19 rate among incarcerated individuals was nearly 3 times that of the general population of Massachusetts and 5 times the rate in the United States	Screening of prisoners by swabs for COVID-19 had a preventive role within prisons
Clarke et al. [26]	Observational cohort study	Ireland	CTT	In the event of a suspected COVID-19 prisoner, the CTT was notified and analyzed all the contacts of this suspected case. All of these prisoners were subjected to both swabs and subsequently quarantine, regardless of the outcome of the swab. If one was positive, the cycle was repeated. If it was negative, the quarantine continued but the cycle of contact tracing was interrupted	CTT was a very effective tool for preventing the risk of COVID-19 infection in prisons
Vest et al. [27]	Observational cohort study	Texas	Reduction of crowding in prisons	85% of the maximum capacity was used as a cut-off for the maximum filling of prisons. This cutoff managed to contain the risk of COVID-19 infection	Reducing prison crowding was able to control the risk of COVID-19 infection
Marco et al. [28]	Retrospective cohort study	Barcelona	Swab analyses	Oral pharyngeal swabs by SARS-CoV-2 PCR analysis were performed on 148 inmates and 36 prison staff. 24.1% of these inmates and personnel tested positive; prisoners were quarantined	Generalized screening, isolation and evaluation of infected persons were key measures. Symptom-based surveillance needs to be complemented by rapid contact-based monitoring to avoid a spread of COVID-19
Marmolejo et al. [29]	Ecological study	Argentina, Chile, Colombia, and Mexico	Reduction of crowding in prisons	Controlled decarceration, limiting new prison admissions, increased use of PPE	Controlled decarceration, limiting new prison admissions, increased use of PPE, decreased the risk of COVID-19 infection
Brinkley-Rubinstein et al. [30]	Observational cohort study	USA	Reduction of prisoner transfers between different prisons	The correlation between the incidence of COVID-19 and prison transfers of inmates was examined. Transfers between prisons positively correlated with the incidence of COVID-19 infection	Limiting inter-prison transfers reduced the risk of COVID-19 infection
Brinkley-Rubinstein et al. [32]	Observational cohort study	USA	Vaccine	2380 residents inside the prison (prisoners and staff) who had received at least one dose of the vaccine were analyzed. Only 27 tested positive for SARS-CoV-2, testifying the vaccine’s effectiveness even in the prison population	The vaccine was an extremely effective tool for the prevention of COVID-19 in prisons

## Data Availability

Data sharing not applicable; no new data were created or analyzed in this study.
